# Species disparity response to mutagenesis of marine yeasts for the potential production of biodiesel

**DOI:** 10.1186/s13068-019-1459-y

**Published:** 2019-05-22

**Authors:** Boutheina Bessadok, Andrea Santulli, Thomas Breuck, Saloua Sadok

**Affiliations:** 1 Blue Biotechnology and Aquatics Bioproducts Laboratory (B3Aqua), Institut National des Sciences et Technologies de la Mer – INSTM-Annexe La Goulette, 60 Port de Pêche, 2060 La Goulette, Tunisia; 2 Institut National Agronomique de Tunisie (INAT), 43 Avenue Charles Nicolle, 1082 Tunis, Tunisia; 3 Consorzio Universitario della Provincia di Trapani (CUPT), Lungomare Dante Alighieri, 91016 Casa Santa, TP Italy; 4 Fachgebiet Industrielle Biokatalyse, IBK Technische Universität München, Lichtenbergstraße 4, 85748 Garching, Germany

**Keywords:** Oleaginous yeast, Lipid, Fatty acid, Biomass, Biodiesel, Mutagenesis optimization, Ethyl methanesulfonate

## Abstract

**Background:**

Among the third-generation biodiesel feed stock, oleaginous marine yeasts are the least studied microorganisms for such purpose.

**Results:**

Wild strains yeasts were isolated from various Tunisian marine sources including fish waste (*Candida tenuis* CtTun15, *Debaryomyces hansenii* DhTun2015, *Trichosporon asahii* TaTun15 and *Yarrowia lipolytica* YlTun15) and seawater (*Rhodotorula mucilaginosa* RmTun15). Following incubation with ethyl methanesulfonate (EMS: 75 mM) for various periods of time (T15, T30, T45, T60 min), the cell viability of these strains responded differentially according to yeast species. For instance, mutated CtTun15 did not survive after 30 min of EMS treatment; higher resistances were observed in DhTun2015 (45 min), in YlTun15, RmTun15 and in TaTun15 (60 min) but with significant decreased cell viabilities (survival rate: 6.02, 3.16, 11.22, 11.58, 7.70%, respectively). For all surviving mutated strains, the optima of biomass and lipid yields were detected after 96 h in YPD culture; but derived from strains submitted to different period of EMS incubation. In most mutated strains, the maximum biomass (BP) and lipid (LP) productivities coincided and were observed after 30 min of EMS incubation. Only CtTun15 showed different optima of BP and LP (after 30 min and 15 min, respectively). The fatty acids (FA) compositions considered essential in the prediction of biodiesel criteria; were highly affected by EMS mutagenesis. Essentially, 30- and 45-min EMS incubation induced the highest levels of PUFA and MUFA in YlTun15, RmTun15 and TaTun15 with non-significant differences in the different times. However, CtTun15 and DhTun2015 mutant strains responded differently, with the highest levels of MUFA observed following 15 and 45 min; and that of PUFA after 30 and 45 min, respectively.

**Conclusion:**

The methyl-esterification of FA from the three mutated yeast strains (30 min—YlTun15, RmTun15 and TaTun15) yielded biodiesel with physical proprieties consistent with the International Standard System. However, investigations are needed for up-scaling biodiesel production.

**Electronic supplementary material:**

The online version of this article (10.1186/s13068-019-1459-y) contains supplementary material, which is available to authorized users.

## Background

Intensive investigations are currently oriented toward the exploration of renewable energy sources as substitutes to fossil fuels [1, 2]. Among such sources, oleaginous microorganisms that can accumulate more than 20% lipids dry weight represent promising substitutes [3]. Moreover, the quality of their lipid allows their potential use in commercial production of oil for food, chemical and energy applications [4–6]. For instance, biodiesel as type of bio-fuels produced mainly by a transesterification process, are used in some European countries in a way similar that of to the mineral diesel and, with some additives, applicable with various engines [7].

*Yarrowia*, *Candida*, *Rhodotorula*, *Rhodosporidium*, *Cryptococcus*, *Trichosporon*, and *Lipomyces* were reported as genera of oleaginous yeast [8, 9] and each are also known as single-cell oil (SCO). Meng [6] reported that some species of *Rhodosporidium*, *Rhodotorula*, and *Lipomyces* are able to accrue lipids to more than 70% of their dry weight. The accumulation of fat in yeast is affected by several parameters that may be grouped as physical (e.g., pH, temperature, light) and chemical (e.g., carbon and nitrogen sources) [10, 11]. However, the TAGs synthesized by oleaginous yeasts consist primarily of C16 and C18, such as C16:0, C16:1, C18:0 C18:1 and 18:2 [12, 13], with varying amounts of shorter (C14) and longer (C26) fatty acid chains, which have key roles in protein modification [14]. The fatty acid (FA) composition of the microbial lipids was found to be similar to vegetable oil, which is commonly used in biodiesel production. Thus, microbial lipids can be used as a potential raw material for biodiesel production [15]. To enhance lipid and FA content in yeast, several techniques were developed including the classical random metagenesis. For such a purpose, ethyl methanesulfonate (EMS) is amongst the most common alkylating agents used in yeast as mutation-inducing agents. Thus, EMS has been used to induce the over-production of metabolites in microalgae, including eicosapentaenoic acid (EPA) [16], astaxanthin, carotenoids [17–20] and hydrogen [21]. In contrast, EMS-random mutagenesis has also been used to develop mutant strains of microalgae devoid of EPA [22].

The aim of this study was to determine the effect of chemical mutagenesis with EMS on the cell viability, and the lipid and biomass productivity of five oleaginous yeasts, *Rhodotorula mucilaginosa* RmTun15, *Y. lipolytica* YlTun15, *Trichosporon asahii* TaTun15, *Debaryomyces hansenii* DhTun2015 and *Candida tenuis* CtTun15. Based on a thorough literature survey and using the fatty acid profile of each of the wild and mutated strains, along with the physical proprieties of Biodiesel, allowed us to predict which of the strain that can produce the best quality of Biodiesel.

## Results and discussion

### Determination of the optimal concentration of EMS

A preliminary study was carried out to determine the optimum of growth for each wild yeast strain as well as the number of colonies formed. During a cultivation period of 144 h in YPD, all species showed an optimum growth after 120 h, but with different values (Table 1). To determinate the effect of mutagenesis, the wild species were subjected to various levels of EMS (25, 75 and 100 mM) in different volumes (15, 50 and 100 µl) during a fixed period (20 min). Using *ANOVA* test, the experimental data were used to identify the specificity of EMS for the different marine organisms and their effects on their growth and viability (Additional file 1: Table S1). As single factors, the concentration and the volume of the mutant agent have significant effects on both colonies numbers counted and growth (*p* < 10^−4^) after 48 and 120 h, respectively, with the number of colonies inversely correlated with the concentration of EMS. The combined effects of both factors were equally proved in all mutated strains as the *F*-value was significantly higher than *p*-value [23].

**Table 1:** Optimal OD and number of colonies formed of different wild species of marine yeasts after 120 h of culture (*n* = 3 for each parameter and for each strain; value = mean ± standard error)

	RmTun15	YlTun15	TaTun15	DhTun2015	CtTun15
OD (600 nm)	24.36 ± 0.37	24.14 ± 0.31	27.36 ± 0.10	21.92 ± 0.84	28.21 ± 0.71
Colony (CFU)	311 ± 11	205 ± 12	247 ± 13	222 ± 10	213 ± 22

The relationship between independent and dependent variables has been illustrated by the three-dimensional (3D) representation (Table 2). To each plot and for each strain, individual contour curve corresponded to an infinity responses of the tested variables combination including their impact on cell viability (number of colony) and growth (OD at 600 nm). Hence, for all strains, the number of colonies decreased with increased EMS concentration and volume. However, the cell growth increased with both rising variables up to 75 mM EMS level than showed significant decrease. As shown in Tables 2 and 3, the lethal doses and volumes for CtTun15 species corresponded, respectively, to 75 mM/100 μl in experiment 1; 100 mM/50 µl in experiment 2; and 100 mM/100 μl in experiment 5. Such lethal doses/volumes were depicted in experiment 5 for the remaining strains but additionally for DhTun15 in experiment 1. In experiment 9, where EMS volume was 50 μl and concentration was 75 mM, all surviving mutated yeast strains presented the best growth. The combination defined in experiment 9 was considered as optimal as it reflected the best growth in all strains, although the high mortality rate was recorded in such conditions indicating the mutagenic efficiency of the procedure as suggested in other study [24].

**Table 2:** Three-dimensional responses plot and regression equations of the models developed for response variables *Z*_Cell_ colonies number formed (CFU); *Z*_Grow_ growth (OD at 600 nm) obtained from 5 species of marine yeast RmTun15

	Colonies number (CFU)	Cell viability (OD 600 nm)
RmTun15	*Z*_Cell_ = 325.649 + 2.119*x* − 0.035*x*^2^ − 3.935*y* + 0.154*y*^2^ + 0.002*xy*	*Z*_Grow_ = − 11.995 + 0.894*x* − 0.006*x*^2^ + 0.449*y* − 0.002*y*^2^ − 0.004*xy*
YlTun15	*Z*_Cell_ = 206.885 + 1.181*x* − 0.021*x*^2^ − 1.874*y* + 0.005*y*^2^ − 0.005*xy*	*Z*_Grow_ = − 0.683 + 0.605*x* − 0.004*x*^2^ − 0.404*y* + 0.002*y*^2^ − 0.003*xy*
TaTun15	*Z*_Cell_ = 89.430 + 12.438*x* − 0.143*x*^2^ − 3.845*y* + 0.016*y*^2^ − 0.006*xy*	*Z*_Grow_ = 9.071 + 0.683*x* − 0.007*x*^2^ + 0.131*y* + 0.002*y*^2^ − 0.001*xy*
DhTun2015	*Z*_Cell_ = 264.903 − 0.148*x* + 0.014*x*^2^ − 2.404*y* + 0.007*y*^2^ − 0.003*xy*	*Z*_Grow_ = 5.523 + 0.177*x* − 0.001*x*^2^ + 0.493*y* + 0.003*y*^2^ − 0.003*xy*
CtTun15	*Z*_Cell_ = 322.879 − 4.192*x* + 0.018*x*^2^ − 2.411*y* + 0.011*y*^2^ − 0.002*xy*	*Z*_Grow_ = 19.612 + 0.436*x* − 0.004*x*^2^ − 0.206*y* + 0.001*y*^2^ − 0.0005*xy*

YlTun15. TaTun15. DhTun2015 and CtTun15 in function of studied factors *x*. concentration of EMS [mM] and *y:* volume of EMS (µl)

**Table 3:** Experimental conditions and results of central composite design (2 3-level factors, 3 blocks, 9 run) with *n* = 3 for each assay

Experiment	Variables		Response									
			YlTun15		TaTun15		RmTun15		DhTun2015		CtTun15	
	EMS [mM]	Volume EMS (ml)	Colony (CFU)	OD (600 nm)	Colony (CFU)	OD (600 nm)	Colony (CFU)	OD (600 nm)	Colony (CFU)	OD (600 nm)	Colony (CFU)	OD (600 nm)
1	75	100	23	19.41	19	21.9	33	19.44	0	0	0	0
2	100	50	21	20.16	29	21.26	31	19.57	5	15.68	0	0
3	25	15	182	20.12	222	21	287	15.38	200	13.95	191	16.36
4	25	50	163	20.47	187	21.9	217	16.12	171	18.12	163	17.68
5	100	100	0	0	0	0	0	0	0	0	0	0
6	75	15	175	20.14	193	21.18	272	20.94	173	19.63	97	24.68
7	100	15	81	18.95	161	19.38	117	16.5	63	14.21	48	23.15
8	25	100	79	19.48	130	20.38	121	18.44	94	20.31	87	20.65
9	75	50	89	21.8	95	22.41	132	22.82	82	21.31	13	25.53

### Effect of EMS on cell growth and biomass productivity

During the last decades, mutagenesis on marine yeast was performed using various agents such as irradiation [25], heavy-ion [26] or UV/EMS/NTG on a random mutation [20]. Studies on marine yeast related to the effect of EMS are scarce and the present work is the first study using such agent following various times of exposure. Using the optimal condition of EMS concentration (75 mM) and volume (50 µl), the effect of EMS on cell growth of the different yeast strains following various time of exposure is shown in Table 4 where codes were added for each strain at each time of EMS exposure. Statistical analysis (*p* < 0.05) showed that each strain reacted differently, but with similar trend of change. Thus, each strain has a specific response that can be summarized with cell death rate that ranged from 13.17 to 88.78%; 12.54 to 88.42% and 21.86 to 92.30%, respectively, for YlTun15, RmTun15 and TaTun15 between 15 and 60 min. For *D. hansenii DhTun2015*, the mortality rate varied between 22.07% at 15 min and 96.84% at 45 min, reaching 100% at 60 min. Regarding *C. tenuis CtTun15*, the total mortality rate was reached at 45 min and increased from 54.46% at 15 min to 93.89% at 30 min. It is worth noting that the effect of EMS is also related to its concentration in the medium [27]. Mobini-Dehkordi et al. [28] found that EMS mutagenesis affects cell viability of the yeast *Saccharomyces cerevisiae* reaching 43% after 45 min of exposure to EMS. Tapia et al. [25] reported that after 40-min irradiation, the colony number of the oleaginous yeast *Lipomyces starkeyi* was reduced to approximately to 5% of the total colonies present in control plates. Ma et al. [26] found that heavy-ion mutagenesis had the same effect on the mortality rate of *Nannochloropsis* that was enhanced from 15 to 89% with increasing irradiation doses. Doan et al. [29] and Anandarajah et al. [30] in EMS mutagenesis for *Nannochloropsis* had also reported similar phenomenon. *Chlorella* cells exposed to 0.28-M EMS showed a survival rate of 50% [31] and Zhang et al. [24] observed that 0.6–0.8 M of EMS was the optimal mutagenic range for *Desmodesmus* sp. S81 and G41 with survival rate of 30.66 ± 0.57% and 39.28 ± 1.13%, respectively, for 30 min of treatment. Nojima et al. [32] found for the strain *Chlorococcum* sp. FFG039 that the treatment with 0.25–0.5-M EMS or MNNG results in a survival rate between 0 and 10%. The effect of EMS on survival rate was also detected in the red algae *P. yezoensis*, the percentage of living cells decreased upon exposure to 1 and 2 mM of EMS to 52 and 10% at day 5, respectively, and was null for higher concentrations of EMS (3 and 4 mM) [33].

**Table 4:** The number of colonies formed (CFU) observed in the control and the yeast strains treated with EMS after different exposure times

Control	YlTun15		RmTun15		TaTun15		DhTun15		CtTun15	
	205 ± 12^a^		311 ± 19^1^		247 ± 13*		222 ± 10^v^		213 ± 22*	
15 min	MY1	178 ± 17^b^	MR1	272 ± 17^2^	MT1	193 ± 7**	MD1	173 ± 4^w^	MC1	97 ± 17**
30 min	MY2	89 ± 15^c^	MR2	132 ± 11^3^	MT2	95 ± 11***	MD2	82 ± 6^x^	MC2	13 ± 6***
45 min	MY3	66 ± 1^d^	MR3	98 ± 7^4^	MT3	49 ± 3****	MD3	7 ± 5^y^	–	–
60 min	MY4	23 ± 3^e^	MR4	36 ± 3^5^	MT4	19 ± 5*****	–	–	–	–

(*n* = 6 in each case. Value = mean ± Standard error), each column includes the designation of the mutated strain at each time

Symbols, letters and numbers refer to statistically significant productivity values (Tukey test. *p* < 0.05)

Asterisks (–) refer to No colony on the plate

**Fig. 1:**
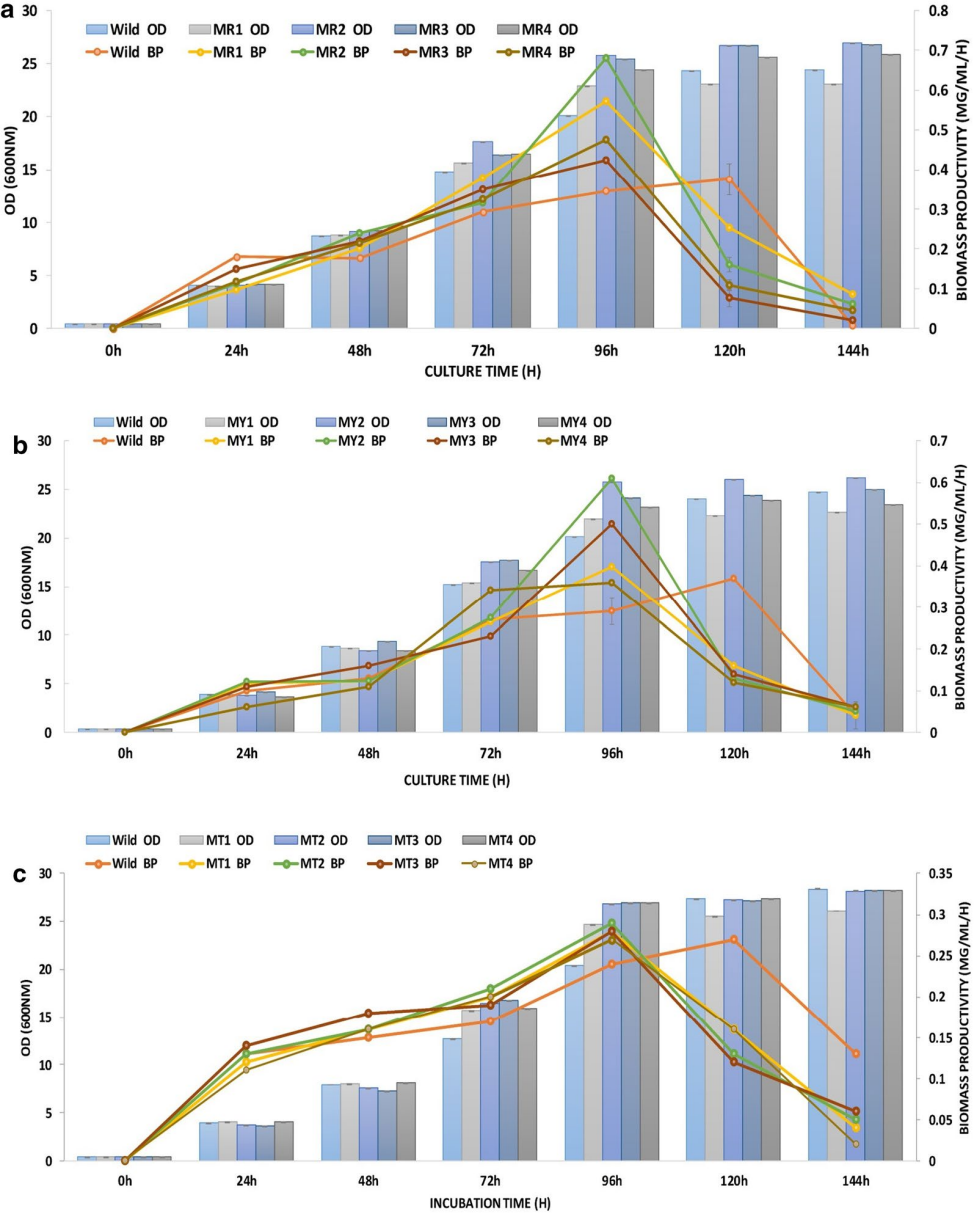

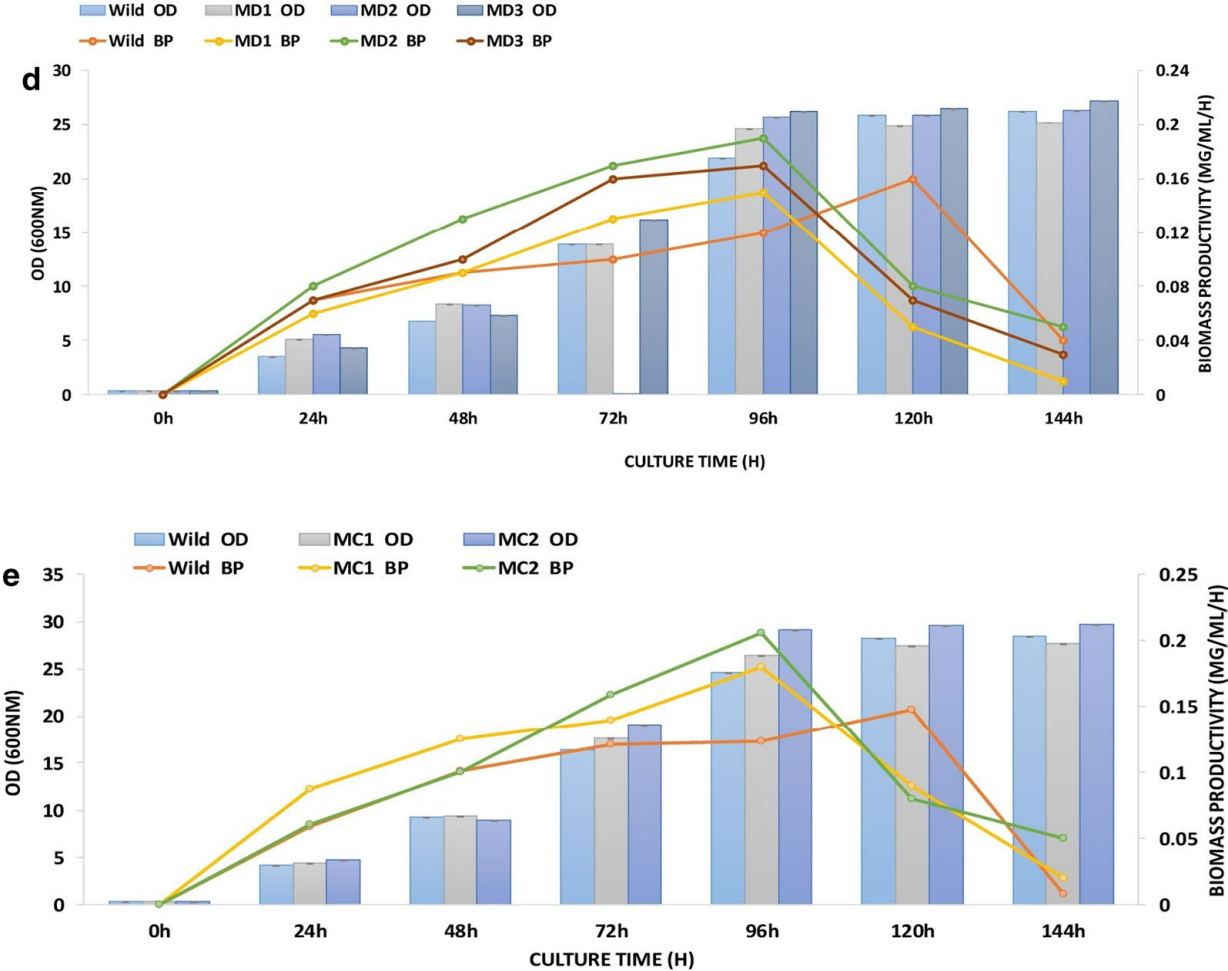
The effect of EMS treatment on the growth (OD) and biomass productivity (BP) of wild and mutated strain of **a** *Rhodotorula mucilaginosa* (RmTun15-Accession Number MF327252); **b** *Yarrowia lipolytica* (YlTun15-Accession Number MF327143); **c** *Trichosporon asahii* (TaTun15-Accession Number KY509046); **d** *Debaryomyces hansenii* (DhTun2015-Accession Number KY5083343) and **e** *Candida tenuis* (CtTun15-Accession Number KY558632). (*n* = 6 in each case, Vertical bar = ± SE)

Figure 1 summarizes the effect of the mutant EMS on the growth and biomass productivity of all studied strains. For all wild species, the optimal yeast productions were obtained after 120 h of YPD culture with a biomass productivity of 0.15; 0.16 and 0.27 mg/ml/h for CtTun15, DhTun2015 and TaTun15. For YlTun15 and RmTun15, the biomass productivity was 0.37 mg/ml/h for both wild strains.

Thus, the optimum growth for all mutated strains was after 96 h of culture, with significantly higher rates than the wild strains. The kinetics monitoring of the biomass productivity suggests that for all mutated strains, the maximum growth coincided with the optimum of the biomass recovered at 96 h. Figure 1a, b showed that BP augmented significantly to reach 0.68 and 0.61 mg/ml/h for MR-2 and MY-2, respectively. For MT-2, MD-2 and MC-2, the biomass productivities were estimated to be 0.29; 0.19 and 0.21 mg/ml/h and which were significantly higher than the wild strain (Fig. 1c–e). The noticeably increased growth rate of mutants may be explained by the fact that some mutants have an enhanced metabolic pathway to improve cell growth. Analogous improvements of growth using random mutagenesis were mentioned to be in various other mutant microorganisms, including *Rhodotorula* sp. [20]; *Ulva fasciata* [34], *Scenedesmus dimorphus* [35]; *Chlamydomonas reinhardtii* [36], *Chlorococcum* sp. FFG039 [32] and the red alga PyE2 [33]. According to Tapia et al. [25], the reduction of cell growth is correlated with the inhibition of lipid biosynthesis metabolism that is stimulated by a different mutant agent such as UV, EMS, and MNNG.

### Effect of EMS on lipid content and productivity

The effect of the treatment with EMS was also detected on the yield of lipid and lipid productivity. The lipid yield (L), lipid content (wt%) and final lipid productivity (LP) are shown in Table 5. Thus, the increase in biomass induced an increase in the lipid yield with a maximum of 3.21 ± 0.09 g/100 g of wet biomass of the strain MC-1. The final lipid productivity increased significantly from 0.022 in wild CtTun15 to 0.027 mg/ml/h in MC-1 and then decreased to 0.024 mg/ml/h in MC-2. Wild strain of *D. hansenii* DhTun2015, MD-1 and MD-2 had a mean yield of 2.19 g/100 g of wet biomass (mean LP 0.019 mg/ml/h) and then decreased significantly to 1.36 ± 0.19 g/100 g wet biomass with a lipid productivity of 0.112 mg/ml/h in MD-3. The wild strain *R. mucilaginosa RmTun15 and Y. lipolytica YlTun15*, known as oleaginous yeasts [37] produced 8.18 ± 0.02 and 10.32 ± 0.21 g/100 g lipid (wet biomass). Among the four mutant strains of RmTun15, MR-2 showed the highest lipid productivity with 0.130 mg/ml/h. This mutant strain showed 11.82 ± 0.33 g/100 g of wet biomass, which was significantly higher than the lipid yield of the wild strain, MR-1, MR-3 and MR-4. The mutant strains showed a higher lipid content than the wild strain (36%) with a maximum of 46%, 38% and 39% for MR-2, MR-3 and MR-4, respectively. An increased lipid yield (13.22 ± 0.11 g/100 g of wet biomass) and a high lipid productivity (0.130 mg/ml/h) were found in MY-2 strain. Concerning TaTun15, a significant rise of 0.82% was noticed in MT-1 when compared to the wild strain. For MT-2, MT-3 and MT-4, however, the mean lipid yield was 5.77 g/100 g (wet biomass) and a mean lipid productivity of 0.058 mg/ml/h. According to Dai et al. [4], *R. glutinis* accumulated lipids up to 49.25% on a cellular biomass basis with biomass yield of 29.77 g/l. Tapia et al. [25] found that for *L. starkeyi* mutated, oleaginous yeast, the lipid content productivity varied from 0.023 ± 0.001 g/l/h to 0.032 ± 0.002 g/l/h, lipid content ranged from 29.88 ± 1.71 to 39.60 ± 1.30%. Doan and Obbard [29] found that for *Nannochloropsis* sp. the mutagenesis with EMS increased the total lipid content of mutant strain from 16.2 ± 2.6% in day 10 to 50.8 ± 6.8% in day 18 representing 1.5 to 2 times higher than that of wild strain (7.6 ± 1.8% in day 10 to 34.0 ± 3.8% in day 18). Kitahara et al. [38] reported that all mutant strains of *Rhodosporidium toruloides* showed higher lipid productivity than the wild type (WT). In this study, the biomass and lipid yield of wild RmTun15 were higher than that for *R. mucilaginosa* [39]. The mutated strain XR-2, obtained by a combined mutagenesis of ARTP and NTG of *R. toruloides,* had an increased lipid and carotenoids productions [40]. The chemical mutant MNNG increased the lipid and the biomass production of *Y. lipolytica* NCIM 3589 as mentioned by Katre et al. [41]. Also, lipid production and the lipid productivity of oleaginous yeast *R. toruloides* were augmented by UV light mutagenesis [42]. In addition, random mutagenesis with UV and selection using cerulenin were effective to improve the lipid productivity of the mutated strain of *Cryptococcus curvatus* NBRC-0732 and *Rhodosporodium toruloides* NBRC 10033 [43]. It was also reported that for *Trichosporon* sp. cultivated in glucose as carbon source, the lipid yield was 21.45 g/l which was much higher than the lipid yield of both wild and mutated TaTun15 as well as the other studied strains either *T. cutaneum* CTM 30125 (2.8 g/l) [44, 45]. Sarayloo et al. [46] increased the lipid productivity of a *Chlorella vulgaris* (UV715-EMS25) mutant strain to 91 mg/l/days; the lipid content and biomass were, respectively, 67% and 35% higher than those of the wild type (WT). It is worth noting that several studies were made including engineering metabolic and phenotypes of strain to increase the biomass and the lipid production [47–53].

**Table 5:** Lipid yield (L), lipid content (LC) and final lipid productivity (LP) in mutated and wild YlTun15; RmTun15; TaTun15; DhTun2015 and CtTun15 (*n* = 9 for each strain ± means with standard error)

	Strain	L (g/100 g)	LC (w%)	LP (mg/ml/h)
YlTun15	Wild	10.32 ± 0.21	42.88 ± 0.14^c^	0.090^b^
	MY-1	11.81 ± 0.08^b^	46.41 ± 1.35^b^	0.099
	MY-2	13.22 ± 0.11^a^	51.74 ± 0.17^a^	0.130^a^
	MY-3	10.22 ± 14	39.68 ± 0.13	0.099
	MY-4	10.26 ± 0.11	41.24 ± 0.84^c^	0.082^c^
RmTun15	Wild	8.18 ± 0.02	36.83 ± 0.21^C^	0.073^E^
	MR-1	11.13 ± 0.16^B^	35.39 ± 0.11^C^	0.113^B^
	MR-2	11.82 ± 0.33^A^	46.98 ± 0.31^A^	0.130^A^
	MR-3	10.63 ± 0.14^C^	38.08 ± 0.41^B^	0.086^C^
	MR-4	9.61 ± 0.27^D^	38.89 ± 0.24^B^	0.080^D^
TaTun15	Wild	3.67 ± 0.44^1^	21.80 ± 1.10^α^	0.037^−^
	Mt-1	4.86 ± 0.08^2^	25.98 ± 0.21^β^	0.049^−^
	Mt-2	5.73 ± 0.46	29.94 ± 0.11	0.059
	Mt-3	5.75 ± 0.10	31.47 ± 0.42	0.058
	Mt-4	5.84 ± 0.10	32.02 ± 0.27	0.059
DhTun2015	Wild	2.32 ± 0.09	19.76 ± 0.81	0.018
	MD-1	2.18 ± 0.08	20.14 ± 0.18	0.020
	MD-2	2.07 ± 0.17	20.57 ± 0.33	0.019
	MD-3	1.36 ± 0.19*	12.25 ± 0.19*	0.011*
CtTun15	Wild	2.53 ± 0.16	25.63 ± 0.85	0.022
	MC-1	3.21 ± 0.09**	30.77 ± 0.61**	0.027**
	MC-2	2.76 ± 0.09	27.82 ± 0.32	0.024

Symbols, letters and numbers refer to productivity values statistically significant within the same species in same parameter (Tukey test* p *< *0.05)*

In this study, the classical mutagenesis technique using ethylmethane sulfonate was applicable to increase lipid content and production of different strain of marine yeast (RmTun15; CtTun15; DhTun2015, TaTun15 and YlTun15) and select mutated strain with a high single-cell oil (SCO) content suitable for Biodiesel production.

### Effect of EMS on FAMES profile

**Fig. 2:**
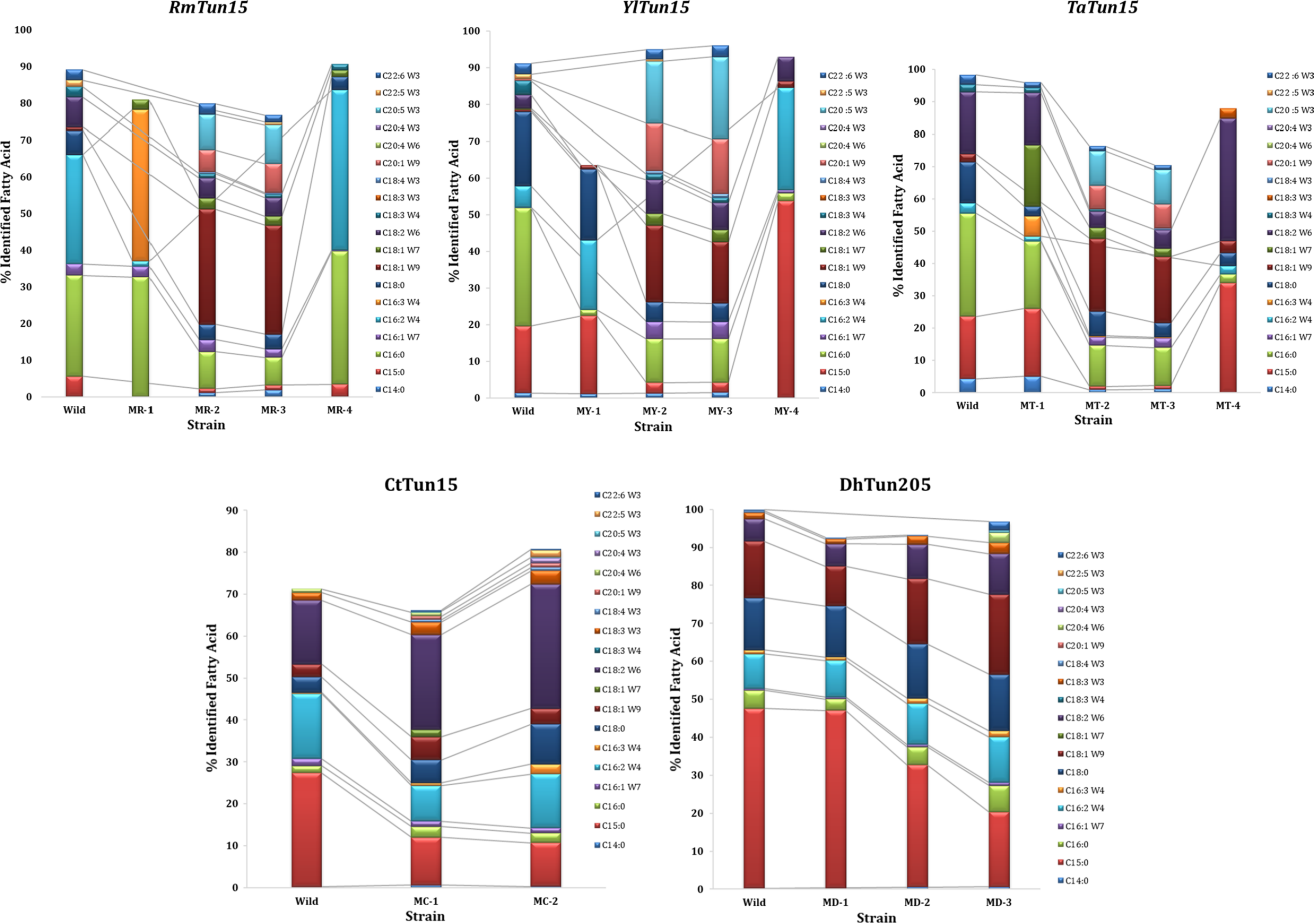
Fatty acids profile (% of identified fatty acid) of wild and mutated strain of RmTun15; YlTun15; TaTun15; CtTun15 and DhTun2015

According to fatty acids methyl-esters (FAMEs) composition, microorganisms can be used in various applications (e.g., nutraceutical) or for Biodiesel production [54, 55]. To evaluate the effect of the chemical mutagenesis using EMS on the quality and the composition of oil, we determine the profile of fatty acid from lipid produced by the wild and mutant strains (Fig. 2). The fatty acid composition of CtTun15-wild strain showed a high content of saturated fatty acid (32.78%), of which C15 accounted for 27% of the total identified FA. Moreover, this strain is rich in PUFA with essentially C16:2 (15.5%) and C18:2 (15.26%). Changes in the FA profile and concentration occurred following mutagenesis. Thus, for MC-1 and MC-2, the C15:0 fraction decreased significantly with an average of 10.87%; while C18:0 increased from 3.74 to 5.54% and 9.45% for MC-1 and MC-2, respectively. The C18:2 represented 22.66% at MC-1 and about 30% at MC-2. The level of MUFA doubled in MC-1 (9.36%) and then decreased to 6% for MC-2, while remaining higher than the control strain (4%). The increase in the PUFA level is dependent on the occurrence and detection of new PUFAs such as C18: 2, C20: 1; C20: 4, C20: 5, C22: 5, and C22: 6. As for RmTun15, the control strain profile is distributed mainly in SFA (38%), mostly C16 (27%) and C18 (6%), and PUFAs (45%), where C16: 2 and C18:2 represent 29 and 8%, respectively, of the total fatty acids. After mutagenesis, the lipid profile changed and there was a significant increase in MUFA levels with an average of 43.53% represented by C18: 1 (30.55%) in MR-2 and MR-3. In the MR-4 strain, the FA composition changed as the wild and MR-1 strains remained statistically different and the percentage of SFA and PUFA increased to 43 and 45%, respectively, whilst MUFA decreased to 2%. The lipid profile analysis of the wild strain DhTun2015 showed that the SFA fraction represented 66% of which C15:0 fatty acid was predominant (47%) and MUFA and PUFA represented 15 and 18%, respectively. After mutagenesis, the saturated fatty acid composition of MD-1 is similar to the wild type (with 47% C15:0). This content decreased significantly in MD-2 and MD-3. The PUFA fraction increased significantly from 18% in wild strain to 20; 23 and 32% in MD-1, MD-2 and MD-3, respectively.

For TaTun15, there was a significant decrease in SFA content in MT-2 (22%) and MT-3 (18%) compared to the wild species with an average of 20%. This decrease is mainly dependent on the fall of C15:0 followed by C16:0 and C18:0. For MT-2 and MT-3, the average of the MUFA and PUFA fractions were 34 and 18% respectively with C22: 5, representing 10% of the total identified PUFA fraction. Concluding with YlTun15, a relatively balanced profile of FA was found in MY-2 and MY-3 with 21% AGS; 40% MUFA and 33% PUFA. Regarding MY-1 and MY-4, a profile similar to that of wild strain was found with the fraction SFA > PUFA > MUFA. The spectacular variation in the fatty acid profile of the different mutant strains can be related to the modification and the regulation of the gene involved in the biosynthesis of fatty acid [46, 56].

Comparing these results with those of other studies, it was found that even the mutated strains possess a profile rich in fatty acid with 18 and 16 carbon atoms as described for *R. mucilaginosa* TJY15a [57]; *C. curvatus* [58], *R. toruloides* Y4 [5]; *S. cerevisiae* [28], *R. toruloides* cultivated in glucose and glycerol [59]; the mutated and wild *Nannochloropsis* sp. [30]; *Chlorella minutissima* [60]; *Mortierella isabellina* [61], *Y. lipolytica* in POME [62], *R. mucilaginosa* [63] and *L. starkeyi* [64]. Khot and Ghosh [65] found a similar result for the strain *R. mucilaginosa* IIPL32. Brar et al. report that the fatty acid profile of wild *Trichosporon* sp. was rich in C16 (25.52%), C18 (12.95%), C18:1 (50.05%) and C18:2 (7.92%) or, for the wild *T. cutaneum*, Guerfali et al. found that the fatty acid profile was mainly C16, C18 and C18:1 with 32%, 9.2% and 51%, respectively [44, 45].

Ethyl methanesulfonate is a suitable mutagen for related purposes [66, 67]. In addition, French et al. [68] reported that EMS is a powerful chemical mutagen and its effect on cells is allied to its concentration in a medium. This study may conclude that chemical mutagenesis with ethyl methane sulfonate affects not only biomass productivity and lipid productivity but also the lipid profile, which guides the application of the strains according to desired, ends (Biofuel, PUFA production, etc.). For a better understanding of lipid metabolism in both mutated and wild strain of RmTun15; DhTun2015; CtTun15; TaTun15 and YlTun15, RNA sequencing and comparison of differential gene expression will be investigated in the future.

### Biodiesel quality

According to international standards, biodiesel must follow some criteria such as ASTM 6751-3 (USA), EN 14214 (Europe) and Bureau of Indian Standard (IS 15607-05) for biodiesel [27]. The fatty acid profile determines the quality of biodiesel and it has been recommended that higher proportions of methyl esters of both C16 and C18 SFAs and MUFAs with reasonable amounts of PUFAs are required for a good quality of Biodiesel [1, 65, 69]. Table 6 shows the characteristics of the biodiesel from the wild RmTun15, YlTun15 and TaTun15 and their mutant strain MR-2, MR-3, MY-2, MY-3, MT-2, MT-3. The choice of such strains was based on their optimum lipid yield and fatty acid profile. Biodiesel proprieties can be classified into different categories with the most important being those related to engines, weather conditions, transport and depositing [70]. The cetane number (CN) was considered as a primary indicator of fuel quality that gives idea on the speed and the compression needed for the ignition. In this study, CN varied significantly from wild to mutant stain. Thus, CN increased from 45.26 ± 2.62 in RmTun15 to mean value of 55 min in MR-2 and MR-3 values that are near to the recommended value of standard fuel. The mean value of CN in MY-2 and MY-3 was 32.65 min which statistically is < wild strain CN (59.53 ± 0.39 min). However, for TaTun15, this parameter increased meaningfully from 50.96 ± 4.36 to 67.15 ± 1.29 in MT-2 and 60.08 ± 0.94 in MT-3. To evaluate the stability to oxidation of biodiesel, the iodine value (IV) was estimated. For RmTun15, MR-2 and MR-3, the mean value of this index was 107.43 ± 2.86 gI_2_/100 g. The iodine value (IV) increased expressively from 48.53 ± 1.54 in the wild strain to 163.22 ± 5.35 gI_2_/100 g in My-2 and MY-3. The wild *T. asahii* TaTun15 and the mutated strain MT-3 have a mean value of 89.66 ± 1.46 gI_2_/100 g that is statistically lower than MT-2 (60.04 ± 0.45 gI_2_/100 g). Regarding the saponification value (SV) or the acid value, the statistical analysis showed that all wild strains (TaTun15, RmTun15 and YlTun15) have a mean value of 213.81 ± 3.47 mg KOH which decreased significantly in mutated MY-2 and 3 (195.19 ± 1.02 mg KOH); MR-2 and 3 (153.23 ± 3.74 mg KOH) and MT-2and 3 (146.78 ± 2.29 mg KOH). This variation can be explained by the modification of the fatty acid composition with an increase of the medium and long-chain fatty acid. The oxidation stability was evaluated for the mutated strain with a mean value of 19.74 ± 1.10 h for MR-2 and MR-3; 20.94 ± 2.26 h for MT-2 and MT-3 and 18.26 ± 1.03 h for MY-2 and MY-3. All mutated strains have a density of 0.85 g/cm^3^ and a kinematic value varying from 3.69 to 4.35 ± 0.09 mm^2^/s that was confirmed to the value fixed in the international standard parameter of Biodiesel. The determination of the physical parameter of biodiesel (CV, IV, SN, HHV, viscosity, oxidation stability) of some genus of oleaginous yeast such as *Rhodotorula* [71–73], *Rhodosporidium* [14, 59, 74, 75], *Cryptococcus* [75], *Debaryomyces* [75], *Yarrowia* [75, 76], *Trichosporon* [44, 45] and *Papiliaterma* [77] predicted the good quality of biodiesel generated by those species. Comparing the results of biodiesel predicted to the different strains of this study, we found a similarity with the genus mentioned in some indices such as the cetane number, the iodine value, the kinematic value and cold filter plugging point which was close to the standard parameter of biodiesel (ASTM D6751, EN14214, Indian Biodiesel IS15607 and the commercial biodiesel). According to Knothe [78], the quality of biodiesel depends on the fatty acid composition of the biolipids. Conferring to Khot and Ghosh [65], high monounsaturated fat content is a good quality of biodegradable product. Dai et al. [4] reported that *R. glutinis* could be a potential strain for biodiesel production according to his lipid profile. Li et al. [57] confirmed that *R. muciloginosa* TJY15a constitute a good feedback for biodiesel production. Arora et al. [59] and Khot and Ghosh [65] reported, respectively, the same conclusion for *C. minutissima* cultivated in optimal condition of lipid productivity (N^*L*^P^*L*^) and for the oleaginous yeast *R. mucilaginosa* IIPL32.

**Table 6:** Biodiesel physical proprieties calculated form FAMES profile obtained from wild and mutated strain of wild RmTun15; MR-2; MR-3; wild TaTun15; MT-2; MT-3; wild YlTun15; MY-2 and MY-3 and its comparison with standard fuel parameters (Indian standard biodiesel. ASTM D651-02 and EN14214) and commercial biodiesel. (*n* = 6 for each strain ± means with standard error)

Fuel parameters	SV (mg KOH)	IV (gI_2_/100 g)	CN (min)	HHV (MJ/Kg)	CFPP (°C)	Ln (kV) (mm^2^/s)	*D* (g/cm^3^)	OS (h)	References
ASTM D6751	–	–	47	–	–	1.9–6	–	–	[60]
EN 14214	–	120 (max)	51	≤5 ≤ 20	–	3.5–5	0.86–0.9	≥ 6	[60]
Indian biodiesel IS15607	–	–	51	–	–	3.5–5	0.86–0.9	–	[70]
Commercial Biodiesel	–	130	51	35	− 5	4.5	0.88	4	[60]
*RmTun15*	216.91 ± 5.33^c^	113.26 ± 3.38^C^	45.26 ± 2.62^Y^	38.99 ± 0.24^xy^	− 14.92 ± 0.37 ^fg^	3.87 ± 0.11	0.95 ± 0.04*	6.87 ± 0.02^α^	This work
*Rmtun15*-*MR2*	159.66 ± 0.84^b^	107.76 ± 1.14^C^	53.01 ± 0.43^ZV^	41.27 ± 0.55^w^	− 7.37 ± 0.31 ^fg^	3.80 ± 0.21	0.85	21.71 ± 2.17^βγ^	This work
*Rmtun15*-*MR3*	148.80 ± 6.63^ab^	101.26 ± 4.07^BC^	57.30 ± 2.56^VW^	41.81 ± 0.33^w^	− 7.62 ± 0.33^f^	3.80 ± 0.64	0.85	17.78 ± 3.32^βγ^	This work
*TaTun15*	211.40 ± 5.02^c^	83.10 ± 0.45^B^	50.96 ± 4.34^Z^	39.52 ± 0.08^yz^	16.40 ± 0.2 ^h^	4.35 ± 0.09	0.85	6.36 ± 0.03^α^	This work
*TaTun15*-*MT2*	151.08 ± 3.42^ab^	60.04 ± 4.05^A^	67.15 ± 1.29	42.43 ± 0.15^w^	1.07 ^g^	3.85	0.85	20.11 ± 1.11^γ^	This work
*TaTun15*-*MT3*	142.49 ± 1.17^a^	96.21 ± 2.47^BC^	60.08 ± 0.94 ^W^	42.14 ± 0.08^w^	− 4.71 ± 0.87 ^fg^	4.03	0.85	21.78 ± 3.41^βγ^	This work
*YlTun15*	213.12 ± 0.06^c^	48.53 ± 1.54^A^	59.53 ± 0.39 ^W^	39.96 ± 0.02^z^	31.97 ± 0.5^i^	4.03 ± 0.01	1.1**	18.81 ± 086^βγ^	This work
*YlTun15*-*MY2*	195.64 ± 1.28^b^	160.13 ± 4.83^D^	33.37 ± 1.13^X^	39.01 ± 0.06^xy^	− 6.80 ± 092^f^	3.46 ± 0.22	0.85	16.88 ± 0.99^β^	This work
*YlTun15*-*MY3*	194.75 ± 0.76^b^	166.31 ± 6.24^D^	31.92 ± 1.49^X^	38.95 ± 0.06^x^	− 3.91 ± 0.55 ^fg^	3.69	0.85	17.86 ± 1.08^βγ^	This work
*Rhodotorula glutinis*	149.9	50.6	71.3	–	23.1	–	–	–	[71]
*Rhodotorula graminis*	–	80.30	57.40	40.10	7.8	4.63	0.87	–	[72]
*Rhodosporidium toruloides*	–	61.4	59.1	39.1	9.81	4.72	0.87	–	[59]
*R. toruloides NBRC 0559*	–	77.99	57.02	40.08	8.27	4.65	0.87	–	[74]
*Cryptococcus music JCM24512*	–	75.33	57.26	40.02	8.76	4.62	0.87	–	[74]
*Rhodosporidium diobovatum*	195.4	78	56.7	–	8.90	4.68	0.87	–	[75]
*Rhodosporodium babjevae*	191.1	78	57.3	–	6.5	–	–	–	[75]
*Yarrowia lipolytica*	185.8	78	58.1	–	1.1	–	–	–	[75]
*Debaryomyces etchellsii*	204.85	69.85	75.14	–	− 0.43	–	–	–	[87]
*Trichosporon* sp.	196	59.28	59.57	–	18.5	–	–	–	[44]
*Trichosporon cutaneum*	204.1	57.9	59.9	–	–	0.86	1.38	–	[45]
*Yarrowia lipolytica NCIM3589*	–	87.7	53.11	49.43	–	4.65	1.09		[76]
*Rhodosporidium diobavatum*	–	68.2	57.8	39.86	0.87	4.72	0.87		[14]
*R. kratochvilovae SY89*	192.30	84.83	55.60	37.63	− 4.66	3.66	0.83	9.94	[73]
*Papilioterma laurentii AM113*	–	81.39	65.63	40.18	7.49	4.26	0.87	–	[77]

## Conclusion

The modification induced by the mutant EMS changed the biomass and the lipid productivity of the strains TaTun15; YlTun15; DhTun2015 CtTun15 and RmTun15. This has made it possible to provide competent strains that can be oriented towards various uses (production of carotenoids, EPA, etc.). The fatty acid compositions confirm the oleaginous proprieties of the identified yeasts that were similar to other oily microbes. In addition, depending on the fatty acid profile and the physical properties of the biodiesel, it was concluded that the mutated strain incubated with EMS (75 mM) for 30 min of *R. mucilaginosa* RmTun15 (MR2), *T. asahii* TaTun15 (MT-2; MT-3) and *Y. lipolytica* YlTun15 (MY-2), constitute a potential for the production of Biodiesel. Further study will be conducted to isolate and identify the genes responsible for the over-production of lipid and fatty acid.

## Methods

### Microorganisms

Five strains were used in this study: *D. hansenii* DhTun2015 (Acc. Num KY5083343.1) and *Y. lipolytica* YlTun15 (Acc. Num MF327143.1) isolated from Tunisian farmed *Dicentrarchus labrax* scales and gills, respectively; *C. tenuis* CtTun15 isolated farmed *Sparus aurata* coproduct Acc. Num KY558632.1); *R. mucilaginosa* RmTun15 isolated from shallow water (La Goulette, Tunisia) (Acc. Num MF327252.1) and *T. asahii* TaTun15 isolated from shrimp’s waste (Acc. Number KY509046.1).

### Chemicals

Chemicals used in this study were: *Yeast Extract* (Biokar Diagnostics, France); *Peptone* (Biokar Diagnostics, France); *Glucose* (Sigma, Germany); *Chlormaphenicol* (Biomatik, USA); *Ethyl Methane Sulfonate* (Sigma, Germany); *Sodium Thiosulfate* (Sigma, Germany); *Sodium Phosphate* (Sigma, Germany); *Sodium Chloride* (Sigma, Germany); *2,6*-*Di*-*tert*-*butyl*-*4*-*methylphenol* (BHT) (Sigma, Germany); *Methanol* (Carlo-Herba, France); *Hydrochloric acid* 37% (Carlo Herba, France); *n*-*Hexane* (VWR chemical, France); *Dichloromethane* (VWR chemical, France); *2,2*-*dimethoxypropane* (Sigma, Germany); *Hydrochloric acid solution* (Sigma, Germany); *Sodium Methoxide solution* (Sigma, Germany) and *Nonadecanoic acid* (Sigma, Germany).

### Culture media

From the plate culture of each strain stored at 4 °C, a loop of cell mass was transferred aseptically to 30-ml YPD media (2% peptone, 2% glucose, 1% yeast extract and 100 ppm chloramphenicol) and then incubated in an orbital shaker (LabWit ZWY-240) at 160 rpm and 30 °C for reactivation. Then, from this preculture, YPD cultures were prepared in 100-ml Erlenmeyer flasks (50-ml working volume) with a starting OD of 0.4 at 600 nm. 100 ppm chloramphenicol was added to the culture medium which was incubated at 26° C with a stirring speed of 160 rpm. Samples were taken at 24-h intervals, in triplicate, to determine cell growth, biomass, and lipid accumulation.

### Chemical mutagenesis with ethyl methane sulfonate (EMS)

The yeast strains were treated with EMS following the method elaborated in Hahn Lab [79, 80]. Briefly, the different species were grown in a YPD medium at 30 °C for 12 h. For each culture, 108 washed cells were placed in 15-ml tubes and suspended in 5-ml phosphate buffer (0.1 M, pH:7). Under the hood, EMS was added to each tube, except the non-mutagenized control, and the cells were incubated on a roller at 30° C for varying time points between 0 min and 1 h. At each time point, 8 ml of sterile 5% sodium thiosulfate was added to inactivate the EMS and to stop the mutagenesis. After centrifugation, the cells were resuspended in distilled water, from which plating was applied on YPD agar for 2–3 days until colonies appeared. Finally, the plate was stored at 4° C to await further analysis. Mutated strains are designed as mentioned in Table 3.

### Experimental design

Optimizing concentration and volume of EMS for the different marine yeast species was carried out using the surface response method and *Statistica 13.3 software*. The following values were adopted for determining dependent and independent variables: x-concentration of EMS varying from 25 to 100 mM; *y*-volume of EMS (ml) from 15 to 100 µl, *z*1-number of colonies forming (CFU) and *z*2-growth measured by the optical density at 600 nm OD (600 nm). In this study, the experiment was planned using the 3**(2-0) full factorial design, 3 blocks, 9 runs in the *Statistica 13.3 software*. Central composite design was used for planning, and 9 experiments were performed for two independent factors as shown in Table 3. According to the presented plan, 9 random experiments were performed for each species with three replicates for each experiment.

### Biomass assessment

Biomass was measured by absorbance and dry biomass [60]. For dry biomass preparation, 1-ml aliquots of each culture was taken and transferred to pre-weighted tubes and pelleted. After 12 h at 80 °C, dry biomass weight was determined by calculating the difference between the final and the initial pre-weighted tube. The absorbance readings were realized by taking 1 ml of aliquots during culture at 24-h intervals, properly diluted with water and measured at 600 nm using a spectrophotometer (LLG-uniSPEC 2 UV/VIS)

$${\text{Biomass productivity}}\left( {\text{BP}} \right) = \left( {\left( {{\text{Final biomass}} - {\text{initial biomass}}} \right)} \right)/{\text{cultivation time}}$$

### Determination of lipid content

The lipids content in the yeast was quantified according to a modified Folch’s method [81] with some modifications. The modification was done during the pretreatment of the strain. The fresh biomass, previously incubated overnight at − 80 °C, was mixed with 5 ml of HCl (2 M), incubated for 20 min in ultrasonic ice bath, and then centrifuged at 10,000 rpm, (4 °C) for 10 min to eliminate cellular debris. The supernatant was mixed with 5 ml of NaCl (0.73%) and 20 ml of Folch solution (dichloromethane/methanol/0.01%BHT), and then centrifuged at 8000 rpm for 10 min at 4 °C. The supernatant was collected into a pre-weighed screw-capped glass tubes and evaporated under vacuum with an Evaporator–concentrator (MiVac, GeneVac, England) at room temperature. The lipid yield was determined according to the formula:

$${\text{Lipid yield }}\left( \% \right) = \, \left( {w_{1} - w_{2} } \right)/w_{B} )*100,$$

where *w*_B_ is the weight of wet biomass; *w*_1_ is the final weight of screw-capped glass tube containing the lipid fraction and *w*_2_ is the initial weight of the screw-capped glass tube.

To follow lipid production in the different strains of yeast, some parameters were followed:

$${\text{Lipid productivity}}\left( {\text{LP}} \right) = \left( {{\text{Biomass productivity}}*{\text{lipid yield }}\left( \% \right)} \right)/100$$

$${\text{Lipid content}}\left( \% \right) = \left( {{\text{Weight of lipid}}/{\text{weight of biomass}}} \right)*100$$

### Fatty acid composition

The methylation of fatty acids was performed to obtain the methyl ester derivatives for analysis by gas chromatography, as described by Griffiths et al. [82]. The lipid fraction was diluted in toluene. To 500 µl of the reaction mix, in screw-capped glass tubes, 50 µl of nonadecanoic acid (C19:0) was added to the reaction as an internal standard. Subsequently, 0.1 ml of 2,2-dimethoxypropane and 1 ml of sodium methoxide were added and the sample was mixed briefly then placed in a shaking incubator (900 rpm) at 80 °C for 30 min. The mixture was cooled for 10 min at room temperature and 1 ml of HCl/methanol was added before repeating the incubation. After cooling, 1 ml of bidistilled water and 0.5 ml of *n*-Hexane for GC were added and the tubes were mixed by vortexing. The upper hexane layer containing FAMEs extract was transferred to vials for GC and kept at − 20 °C until analysis. The analysis of the fatty acids was carried out by GC HP model 6890 equipped with an INNOWAX polar capillary column (30 m in length and 0.25 μm in diameter), a Flame Ionization Detector (FID) and an injector splitted. The volume of injection was 1 μl. Fatty acids were identified by comparing the retention times of FAME with SUPELCO™ 19-component FAME mixture (PUFA no. 3, Sigma-Aldrich). Three replicate GC analyses were performed.

### Determination of biodiesel quality

Biodiesel properties were determined using the following formulae, as described by [60, 83–86]:

$${\text{Iodine value:\,IV}}\left( {{\text{gI}}_{2} /100\,g} \right) = \sum 245{\text{DB}}*\% {\text{FC}}/M$$

$${\text{Saponification value:}}\,{\text{SV }}\left( {\text{mg KOH}} \right) = \left( {\sum 560*\left( {\% {\text{FC}}} \right)} \right)/M$$

$${\text{Cetane number:}}\,{\text{CN}} = 46.3 + 5458/{\text{SV}} - \left( {0.255*{\text{IV}}} \right)$$

$${\text{Degree of unsaturation:}}\,{\text{DH }}\left( {\% {\text{wt}}} \right) = {\text{MUFA}} + \left( {2*PUFA} \right)$$

$${\text{Long chain saturation factor:}}\,{\text{LCSF }}\left( {\% {\text{wt}}} \right) = \left( {0.1*C16} \right) + \left( {0.5*C18} \right)$$

$${\text{High heating value:}}\,{\text{HHV }}\left( {{\text{MJ}}/{\text{Kg}}} \right) = 49.43{-}0.041\left( {\text{SV}} \right){-}0.015\left( {\text{IV}} \right)$$

$${\text{Cold flow plugging propriety:\,CFPP}}\, ({^\circ{\text{C}}}) = \left( {3.417*{\text{LCSF}}} \right){-}16.477$$

$${\text{Kinematic viscosity:}}\,{\text{Ln}}\left( {\text{kV}} \right)\left( {{\text{mm}}^{2} /{\text{s}}} \right) = - 12.503 + 2.496*{\text{Ln}}\left( {\sum {\text{M}}} \right){-}0.178*\sum {\text{DB}}$$

$${\text{Density:}}\,D \, \left( {{\text{g/cm}}^{3} } \right) = 0.8463 + 4.9 /\sum M + 0.0118*\sum {\text{DB}}$$

$${\text{Oxidative stability:}}\,{\text{OS }}\left( {\text{h}} \right) = 117.9295/\left( {{\text{wt}}\% {\text{C}}18:2 + {\text{wt}}\% {\text{C}}18:3} \right) + 2.5905$$

with *M* is the molecular mass of each fatty acid component, DB is the number of double bonds, FC is the % of each fatty acid component, MUFA is the weight % of monounsaturated fatty acids and PUFA is the weight % of poly unsaturated fatty acid.

### Statistical analysis

Data were subjected to Analyses of Variance at the 5% level using *SPSS 24.0* software and the *Tukey* test was performed to separate differences among means.

## Additional file

**Additional file 1: Table S1.** Analysis of variance (ANOVA)of the experiment *(p *= *0.05).* (*SS:* sum of squares, *dF:* degrees of freedom, *MS:* mean square, *F*: statistics, *p*: Probability). Summarizes the results of fitting the models of the experimental design to the data by the F test and p value.

## Data Availability

The datasets used and/or analyzed during the current study are available from the corresponding author on reasonable request.

## Abbreviations

ARTP: atmospheric and room temperature plasma
B: biomass
BP: biomass productivity
EMS: ethyl methanesulfonate
EPA: eicosapentaenoic acid
FA: fatty acid
FAME: fatty acid methyl ester
IV: iodine value
L: lipid yield
LP: final lipid productivity
MNNG: *N*-methyl-*N*′-nitro-*N*-nitrosoguanidine
MUFA: monounsaturated fatty acid
PUFA: polyunsaturated fatty acid
SCO: single cell oil
SFA: saturated fatty acid
SV: saponification value
WT: wild type
YPG: yeast–peptone–glucose
