# Correction to: Species disparity response to mutagenesis of marine yeasts for the potential production of biodiesel

**DOI:** 10.1186/s13068-019-1504-x

**Published:** 2019-06-20

**Authors:** Boutheina Bessadok, Andrea Santulli, Thomas Brück, Saloua Sadok

**Affiliations:** 1 Blue Biotechnology and Aquatics Bioproducts Laboratory (B3Aqua), Institut National des Sciences et Technologies de la Mer – INSTM-Annexe La Goulette, 60 Port de Pêche, 2060 La Goulette, Tunisia; 2 Institut National Agronomique de Tunisie (INAT), 43 Avenue Charles Nicolle, 1082 Tunis, Tunisia; 3 Consorzio Universitario della Provincia di Trapani (CUPT), Lungomare Dante Alighieri, 91016 Casa Santa, TP Italy; 4 Fachgebiet Industrielle Biokatalyse, IBK Technische Universität München, Lichtenbergstraße 4, 85748 Garching, Germany

## Correction to: Biotechnol Biofuels (2019) 12:129 10.1186/s13068-019-1459-y

The original version of the article [1] unfortunately contained a mistake in author’s second name. The name of the author has been corrected from Thomas Breuck to Thomas Brück in this correction article. The original article has been corrected.
