# Supplementary material for: Species disparity response to mutagenesis of marine yeasts for the potential production of biodiesel
Source: Biotechnol Biofuels. 2019 May 22;12:129. doi: 10.1186/s13068-019-1459-y (PMC6530083; doi:10.1186/s13068-019-1459-y)
Supplement: Supplementary file 1 — Additional file 1: Table S1. Analysis of variance (ANOVA)of the experiment (p = 0.05). (SS: sum of squares, dF: degrees of freedom, MS: mean square, F: statistics, p: Probability). Summarizes the results of fitting the models of the experimental design to the data by the F test and p value. [file 13068_2019_1459_MOESM1_ESM.docx]

***Additional File 1:***

***Title of data:***

**Table S1:** Analysis of variance (ANOVA)of the experiment *(p=0.05).* (*SS:* sum of squares, *dF:* degrees of freedom, *MS:* mean square, *F*: statistics, *p*: Probability)

***Description of data:***

Table S1 summarizes the results of fitting the models of the experimental design to the data by the F test and p value

|  |  | Growth (OD 600nm) | | | | | Colonies number (CFU) | | | | |
| --- | --- | --- | --- | --- | --- | --- | --- | --- | --- | --- | --- |
|  |  | SS | df | MS | F | *p* | SS | df | MS | F | *p* |
| CtTun15 | Model *_Corr_* | 2944,780 | 8 | 363,0980 | 2,04E+30 | <0,001 | 126832,667 | 8 | 15854,083 | 176,811 | <0,001 |
|  | EMS [mM] (1) | 582,362 | 2 | 291,1810 | 1,62E+31 | <0,001 | 84884,667 | 2 | 42442,333 | 473,335 | <0,001 |
|  | Volume EMS (µl) (2) | 948,282 | 2 | 474,1410 | 2,63E+30 | <0,001 | 36080,667 | 2 | 18040,333 | 201,193 | <0,001 |
|  | 1*2 | 1414,136 | 4 | 353,5340 | 1,96E+30 | <0,001 | 5867,333 | 4 | 1466,833 | 16,359 | <0,001 |
|  | Error | 0,000 | 18 | 1,80E-28 |  |  | 1614,000 | 18 | 89,667 |  |  |
|  | Total | 8410,381 | 27 |  |  |  | 254112,000 | 27 |  |  |  |
|  | Total *_Corr_* | 2944,780 | 26 |  |  |  | 128446,667 | 26 |  |  |  |
|  |  | R^2^=1; R^2^_Adj_=1 | | | | | R^2^=0,987; R^2^_Adj_ =0,982 | | | | |
| DhTun2015 | Model *_Corr_* | 1607,783 | 8 | 200,973 | 8,05E+29 | <0,001 | 147018,174 | 8 | 18377,259 | 457,736 | <0,001 |
|  | EMS [mM] (1) | 252,925 | 2 | 126,463 | 5,07E+29 | <0,001 | 69525,852 | 2 | 34762,926 | 865,866 | <0,001 |
|  | Volume EMS (µl) (2) | 673,258 | 2 | 336,629 | 1,35E+30 | <0,001 | 66379,185 | 2 | 33189,593 | 826,678 | <0,001 |
|  | 1*2 | 681,600 | 4 | 170,400 | 6,83E+29 | <0,001 | 11113,037 | 4 | 2778,259 | 69,200 | <0,001 |
|  | Error | 0,000 | 18 | 2,50E-28 |  |  | 722,667 | 18 | 40,148 |  |  |
|  | Total | 6668,018 | 27 |  |  |  | 354547,000 | 27 |  |  |  |
|  | Total *_Corr_* | 1607,783 | 26 |  |  |  | 147740,741 | 26 |  |  |  |
|  |  | R^2^=1; R^2^_Adj_=1 | | | | | R^2^=0,995; R^2^_Adj_:0,993 | | | | |
| TaTun15 | Model *_Corr_* | 1232,412 | 8 | 154,051 |  |  | 165666,741 | 8 | 20708,343 | 252,427 | <0,001 |
|  | EMS [mM] (1) | 355,879 | 2 | 177,939 |  |  | 63544,852 | 2 | 31772,259 | 387,292 | <0,001 |
|  | Volume EMS (µl) (2) | 329,966 | 2 | 164,983 |  |  | 92233,852 | 2 | 46116,926 | 562,148 | <0,001 |
|  | 1*2 | 546,567 | 4 | 136,642 |  |  | 9888,370 | 4 | 2472,093 | 30,134 | <0,001 |
|  | Error | 0,000 | 18 | 0,000 |  |  | 1476,667 | 18 | 82,037 |  |  |
|  | Total | 10970,294 | 27 |  |  |  | 525139,000 | 27 |  |  |  |
|  | Total *_Corr_* | 1232,412 | 26 |  |  |  | 167143,407 | 26 |  |  |  |
|  |  | R^2^=1; R^2^_Adj_=1 | | | | | R^2^=0,991; R^2^_Adj_ =0,987 | | | | |
| YlTun15 | Model *_Corr_* | 1089,465 | 8 | 136,1830 | 9,19E+06 | <0,001 | 113986,741 | 8 | 14248,343 | 134,090 | <0,001 |
|  | EMS [mM] (1) | 312,715 | 2 | 156,3570 | 5,80E+08 | <0,001 | 50260,519 | 2 | 25130,259 | 236,499 | <0,001 |
|  | Volume EMS (µl) (2) | 324,687 | 2 | 162,3430 | 1,06E+07 | <0,001 | 56943,185 | 2 | 28471,593 | 267,945 | <0,001 |
|  | 1*2 | 452,063 | 4 | 113,0160 | 1,10E+07 | <0,001 | 6783,037 | 4 | 1695,759 | 15,959 | <0,001 |
|  | Error | 0,000 | 18 | 1,48E-05 | 7,63E+06 |  | 1912,667 | 18 | 106,259 |  |  |
|  | Total | 9681,209 | 27 |  |  |  | 340215,000 | 27 |  |  |  |
|  | Total *_Corr_* | 1089,465 | 26 |  |  |  | 115899,407 | 26 |  |  |  |
|  |  | R^2^=1; R^2^_Adj_=1 | | | | | R^2^=0,983; R^2^_Adj_ =0,976 | | | | |
| RmTun15 | Model *_Corr_* | 1030,463 | 8 | 128,808 | 1,53E+31 | <0,001 | 263602,667 | 8 | 32950,333 | 388,328 | <0,001 |
|  | EMS [mM] (1) | 341,820 | 2 | 170,910 | 2,03E+31 | <0,001 | 115232,000 | 2 | 57616,000 | 679,019 | <0,001 |
|  | Volume EMS (µl) (2) | 210,179 | 2 | 105,089 | 1,25E+31 | <0,001 | 135766,222 | 2 | 67883,111 | 800,019 | <0,001 |
|  | 1*2 | 478,463 | 4 | 119,616 | 1,42E+31 | <0,001 | 12604,444 | 4 | 3151,111 | 37,137 | <0,001 |
|  | Error | 1,52E-28 | 18 | 8,42E-30 |  |  | 1527,333 | 18 | 84,852 |  |  |
|  | Total | 8352,531 | 27 |  |  |  | 754778,000 | 27 |  |  |  |
|  | Total *_Corr_* | 1030,463 | 26 |  |  |  | 265130,000 | 26 |  |  |  |
|  |  | R^2^=1; R^2^_Adj_=1 | | | | | R^2^=0,994; R^2^_Adj_ =0,992 | | | | |
